# In Vitro Release, Mucosal Permeation and Deposition of Cannabidiol from Liquisolid Systems: The Influence of Liquid Vehicles

**DOI:** 10.3390/pharmaceutics14091787

**Published:** 2022-08-26

**Authors:** Peera Tabboon, Thaned Pongjanyakul, Ekapol Limpongsa, Napaphak Jaipakdee

**Affiliations:** 1 Division of Pharmaceutical Technology, Faculty of Pharmaceutical Sciences, Khon Kaen University, Khon Kaen 40002, Thailand; 2 Center for Research and Development of Herbal Health Products, Khon Kaen University, Khon Kaen 40002, Thailand; 3 College of Pharmacy, Rangsit University, Pathumthani 12000, Thailand

**Keywords:** cannabinoids, nonvolatile solvent, volatile solvent, surfactants, permeation enhancement, permeation retardation

## Abstract

This work investigated the influence of liquid vehicles on the release, mucosal permeation and deposition of cannabidiol (CBD) from liquisolid systems. Various vehicles, including EtOH, nonvolatile low- and semi-polar solvents, and liquid surfactants, were investigated. The CBD solution was converted into free-flowing powder using carrier (microcrystalline cellulose) and coating materials (colloidal silica). A physical mixture of the CBD and carrier–coating materials was prepared as a control. The non-crystalline state of CBD in the liquisolid systems was confirmed using XRD, FTIR and SEM studies. The CBD liquisolid powder prepared with volatile and nonvolatile solvents had a better CBD release performance than the CBD formed as the surfactant-based and control powders. The liquisolid systems provided the CBD permeation flux through porcine esophageal mucosa ranging from 0.68 ± 0.11 to 13.68 ± 0.74 µg·cm^−2^·h^−1^, with the CBD deposition levels of 0.74 ± 0.04 to 2.62 ± 0.30 μg/mg for the dry mucosa. Diethylene glycol monoethyl ether showed significant CBD permeation enhancement (2.1 folds) without an increase in mucosal deposition, while the surfactants retarded the permeation (6.7–9.0 folds) and deposition (1.5–3.2 folds) significantly. In conclusion, besides the drug release, liquid vehicles significantly influence mucosal permeation and deposition, either enhanced or suppressed, in liquisolid systems. Special attention must be paid to the selection and screening of suitable liquid vehicles for liquisolid systems designed for transmucosal applications.

## 1. Introduction

Orotransmucosal drug delivery is an alternative non-invasive administration route that avoids gastrointestinal decomposition and hepatic first-pass metabolism when achieving systemic drug circulation. The high permeability and vascularization of oral mucosa, especially sublingual mucosa, can offer a rapid onset of the therapeutic action. Drug administration through the oral cavity is fairly simple and convenient, and it allows for self-administration [1]. Unfortunately, only a limited range of compounds with high lipophilicity and water solubility can successfully be delivered transmucosally due to the barrier properties of mucosa tissue. For lipophilic drugs, their permeation through the mucosa may be limited by their poor aqueous solubility and poor dissolution in the oral cavity fluids, as only dissolved or molecularly dispersed compounds are capable permeation [1,2]. Hence, pharmaceutical approaches to overcoming these solubility limitations should be adequately applied.

Cannabidiol (CBD) is one of the lipophilic model compounds (calculated log n-octanol–water partition coefficient (K_ow_) = 8) with limited water solubility (5 ppm) [3,4]. CBD is a non-psychotropic phytocannabinoid originating from the Cannabis species. Its utilization has received considerable attention over the last two decades due to its wide range of pharmacological activities, which include antiepileptic, anticonvulsant, antianxiety, antipsychotic, sedative, antiemetic, analgesic, and anti-inflammatory functions, as well as antioxidant and neuroprotective effects. The oral bioavailability of CBD, as with other phytocannabinoids, is generally low and inconsistent, owing to their limited water solubility and considerable first-pass metabolism [4,5,6]. Transmucosal delivery of CBD through the oral mucosa is considered a suitable alternative to systemic oral delivery in order to avoid the gastrointestinal tract and hepatic metabolism. A recent study revealed that CBD in a readily dissolved form with ethanol-propylene glycol (1:1) was able to slowly permeate through the oral mucosa, whereas its deposition in the mucosal membrane further acted as a CBD reservoir after the CBD delivery device removal [7]. Nonetheless, the long-term use of products with a high content of low-polarity solvents, such as ethanol and propylene glycol, has been associated with mucosa irritation and bad taste, which influence patient compliance and treatment adherence. As in the case of the commercially available oromucosal spray (Sativex^®^), which contains CBD (25 mg/mL) in combination with delta-9-tetrahydrocannabinol (27 mg/mL) dissolved in peppermint oil–propylene glycol–ethanol (~50% *v*/*v* ethanol), up to 25% of patients experienced mouth ulcerations, lesions, pain and soreness at the application site and oral cavity after continuous use [8,9]. A small number of CBD transmucosal formulations, either alone or in combination with other cannabinoids with or without dissolution enhancement strategies, have been documented in the literature and patents [6,10,11].

It is known that pharmaceutical formulations play substantial roles in the improvement of solubility and stability, along with the uniform delivery of active ingredients. Among the recent pharmaceutical approaches toward enhancing the solubility and dissolution of CBD and other cannabinoids [3,4,10,12,13], the liquisolid technique is considered to be a promising and relatively simple strategy that could successfully enhance the dissolution while preserving the stability of cannabinoids [3]. The liquisolid technique refers to a dissolution enhancement system involving the use of liquid vehicles to dissolve or disperse the poor water-soluble hydrophobic active ingredient before converting it into a visibly dry and flowable powder. The resulting liquisolid powder can be further manufactured into several solid dosage forms [14,15].

One of the critical parameters required for the liquisolid technique to be an effective dissolution enhancement system is the liquid vehicles. Nonvolatile liquid vehicles, including propylene glycol (PG), polyethylene glycol 400 (PEG), polyoxyethylene sorbitan monolaurate (P20), etc., are widely and conventionally used in liquisolid systems. However, the use of volatile vehicles, namely, ethyl alcohol (EtOH) and acetone, alone or in conjunction with nonvolatile liquids, have gained increasing interest. The comparable efficacy of EtOH to nonvolatile vehicles in regard to the dissolution enhancement of hydrophobic compounds such as cannabinoids has been recently reported [3]. These liquid vehicles play important roles that involve the dissolution improvement via probable mechanisms, including an enhanced available surface area, enhanced wettability, and/or enhanced water solubility of the hydrophobic compounds [14]. Additionally, the significant intestinal permeation enhancement by liquisolid systems is also associated with the actions of liquid vehicles, either indirectly through the dissolution enhancement and/or directly through the permeation enhancement capacity of the vehicles. Different liquid vehicles yield different enhancement ratios in terms of both dissolution and intestinal permeation [16,17,18].

To our knowledge, no study has yet examined the mucosal permeation and deposition of lipophilic compounds in liquisolid systems. Therefore, in this investigation, the effects of liquid vehicles on the in vitro release, as well as ex vivo mucosal permeation and deposition, of CBD in CBD liquisolid systems were assessed. Different liquid vehicles, namely EtOH, the volatile vehicle, and several nonvolatile vehicles (diethylene glycol monoethyl ether (DEGEE), PG, oleoyl macrogolglycerides (OM) and caprylocaproyl macrogolglycerides (CM)) were investigated. A control CBD powder based on the physical mixture (PM) of CBD isolate with the carrier and coating materials was also prepared. Microcrystalline cellulose (MCC) and colloidal silicon dioxide (CSD) at an excipient ratio (R ratio) of 10:1 were used as a carrier–coating system. MCC and CSD are some of the most frequently employed carriers and coating materials in liquisolid systems. Their properties and validity in liquisolid systems have been widely publicized [3,14,15,16,19].

## 2. Materials and Methods

### 2.1. Materials

Cannabidiol (CBD, CBD isolate, 99%) was kindly provided by the Medicinal Cannabis Research Institute, College of Pharmacy, Rangsit University (Pathumthani, Thailand). Microcrystalline cellulose (MCC; Avicel^®^ PH102) and colloidal silicon dioxide (CSD, Aerosil^®^ 200) were obtained from Onimax Co., Ltd. (Bangkok, Thailand) and Maxway Co., Ltd. (Bangkok, Thailand), respectively. Absolute ethyl alcohol (EtOH) was purchased from QRëC (Auckland, New Zealand). Caprylocaproyl macrogolglycerides (CM, Labrasol^®^, Gattefossé SAS (Saint-Priest, French)), diethylene glycol monoethyl ether (DEGEE, Transcutol^®^ P, Gattefossé SAS (Saint-Priest, French)) and oleoyl macrogolglycerides (OM, Labrafil^®^ M 1944 CS, Gattefossé SAS (Saint-Priest, French)) were provided by Rama Production, Co., Ltd. (Bangkok, Thailand). Glycerin and propylene glycol (PG) were sourced from RCI Labscan Ltd. (Bangkok, Thailand). Polyethylene glycol 400 (PEG) and polyoxyethylene sorbitan monolaurate (Polysorbate 20, P20) were provided by Sigma-Aldrich (Missouri, MO, USA) and AppliChem GmbH (Darmstadt, Germany), respectively. Formic acid was supplied by KemAus (Cherrybrook, Australia). HPLC grade methanol and acetonitrile were supplied by Fisher^®^ Scientific (Loungborough, UK). All chemicals were used as received.

### 2.2. CBD Solubility

The solubility of CBD in the investigated liquids was examined by adding the excess mass of CBD into 1 g of each test liquid in a test tube. The mixture was sonicated (1 h) using an ultrasonicator (Model LUC-405, Daihan Labtech Co., Ltd., Namyangju-si, Korea) and agitated constantly (37 ± 0.5 °C, 24 h) using a shaking water bath (Model LSB-030S, Daihan Labtech Co., Ltd., Namyangju-si, Korea), and the supernatant was filtered through a 0.45 μm syringe filter. The obtained filtrate was weighed, diluted and quantified by HPLC assay. The CBD solubility in the terms of the mg of CBD per g of liquid was calculated. 

### 2.3. Liquid Load Factor (L_f_) of the CBD Solutions

The amount of MCC–CSD required to convert the solution of CBD, 20% *w*/*w*, into a flowable powder was examined using the method previously reported [3,19,20]. In brief, one gram of CBD solution was blended with one gram of MCC–CSD mixture in a glass mortar for 10 min. The resulting CBD admixture was further added and thoroughly blended with MCC–CSD powder in increments of 0.1 g. The addition of, and blending with, the MCC–CSD powder were continued until the CBD powder admixture appeared as a lump-free powder, having the angle of slide of ≤33° and flow rates of ≥6 cm^3^/s. The liquid load factor (L_f_) of the CBD solution, represented as the weight ratio of CBD solution and the amount of MCC in the MCC–CSD powder needed to bring about an acceptable free-flowing CBD liquisolid powder, was computed.

### 2.4. Preparation of the CBD Liquisolid Powder

Five formulations of the CBD liquisolid powder based on five different liquids, namely CBD–EtOH, CBD–DEGEE, CBD–OM, CBD–PG, and CBD–CM, were fabricated by dissolving 1 g of CBD isolate powder with 4 g of liquid vehicle. The CBD solution was then thoroughly blended with 34.55 g of MCC in a mortar for 5–10 min. Afterward, 3.46 g CSD was added and lightly triturated with the CBD–vehicle–MCC admixture. For the CBD–EtOH formulation, EtOH was evaporated, and the CBD–EtOH liquisolid powder was dried out using the hot air oven (45 °C, 30 min) (drying oven SLW 115 STD, POL-EKO-APARATURA sp.j., Wodzislaw Slaski, Poland). The obtained CBD liquisolid powder was packed in an anti-static plastic-coated aluminum foil bag and kept in a desiccator for further evaluation. A physical mixture (PM) powder of the CBD with MCC and CSD at an equivalent quantity to the CBD liquisolid powder was also prepared as the control powder.

### 2.5. Evaluation of the CBD Liquisolid Powder

#### 2.5.1. Flowability

The flowability of the CBD liquisolid systems was investigated via three variables, namely, the flow rate, angle of repose and angle of slide, using the methods described elsewhere [3,21]. The flowability tester BEP2 (Copley Scientific Limited, Nottingham, UK) was used to determine the flow rate and angle of repose. The angle of slide refers to the angle formed between the horizontal plane and the polished metal plate that is observed in the CBD liquisolid powder when it is about to slide. The investigated powder (4 g) was put onto the liftable side of a metal plate, and this side was slowly lifted until the powder began to slide. The angle formed by the plate with a horizontal surface was determined and noted as the angle of slide.

#### 2.5.2. Compatibility, X-ray Diffraction (XRD) and Morphological Investigations

The compatibility of the CBD with the liquid vehicles as well as the MCC–CSD was investigated by means of the attenuated total reflection Fourier-transform infrared (ATR-FTIR) spectra. The ATR-FTIR analysis over a frequency range of 4000–600 cm^−1^ was recorded on the ATR module of a BRUKER TENSOR 27 Fourier-transform infrared spectrometer (Bruker Corporation, Billerica, MA, USA). 

The physical state of the CBD in the CBD liquisolid formulations was investigated using the D8 ADVANCE diffractometer (Bruker BioSpin, Billerica, MA, USA). The XRD patterns of the CBD liquisolid powders, as well as CBD isolate, MCC, CSD and PM powder, were recorded over a 2θ range of 5–40°. 

The morphological characteristics of the CBD liquisolid systems, as well as those of CBD isolate and PM powder, were assessed using a Helios NanoLab G3 CX focused ion beam field-emission scanning electron microscopy (FIB/FESEM) (Thermo Fisher Scientific, Waltham, MA USA). The investigated powder was firstly mounted onto a metallic stub with conductive adhesive tape and then gold sputter-coated to a thickness of approximately 30–50 nm under vacuum conditions. The micrograph was captured under a secondary electron imaging mode at a 10 kV accelerated voltage.

#### 2.5.3. In Vitro Release

The in vitro release under sink conditions was assessed using the method of Aodah et al. [22] with modifications. A modified Franz diffusion cell with a diffusion area of 2.01 cm^2^ and receptor volume of 14.0 ± 0.2 mL was used. Porous synthetic membranes, the Whatman^®^ Nuclepore™ polycarbonate hydrophilic membrane (0.2 µm pore size, Ø 25 mm (Whatman, Piscataway, NJ, USA)), were utilized as the diffusional membranes. The hydrated membrane was mounted between the donor and receiver compartments with a clamp. A receiver chamber with a magnetic stirrer was filled with 50% *v*/*v* ethanol, a receiver medium, and the hydrodynamics were maintained by stirring with a stirrer at 80 rpm (digital magnetic stirrer, VELP SCIENTIFICA., Italy). The temperature was set at 37 ± 0.5 °C. Then, 10 mg of CBD in the form of the CBD liquisolid systems or the PM powder system was placed in the donor compartment chamber with 1.5 mL of simulated saliva fluid (SSF) with a pH of 6.8 [23]. The donor compartment was tightly covered with paraffin film. At predetermined times (0, 0.5, 1, 2, 3, 4, 5 and 6 h), a 0.8 mL aliquot was collected through a sampling port from the receiver compartment, and an equal volume of pre-warmed fresh medium was quickly replenished. The amount of diffused CBD was quantified by HPLC assay. 

*Diffusion data analysis:* The cumulative amount of diffused CBD per area was calculated and plotted against the time. Diffusion data obtained were fitted with zero-order (Equation (1)) and Higuchi (Equation (2)) equations. The diffusion efficiency, expressed as the rectangle area percentage determined from the area under the diffusion curve up to 6 h, were calculated (Equation (3)):

(1) $$M_{t}=M_{0}+K_{0}\cdot t$$

(2) $$M_{t}=M_{0}+K_{H}\cdot t^{1/2}$$

(3) $$\mathrm{Diffusion}\;\mathrm{efficiency}=\frac{\int_{0}^{t}y\times \mathrm{dt}}{y_{100}\times t}$$

where *M_t_* is the amount of CBD released at time *t* and *M*_0_ is the initial amount of the CBD in the receiver medium, which was often zero. *K*_0_ and *K_H_* are the zero-order and Higuchi rate constants, respectively [24].

#### 2.5.4. Ex Vivo Permeation and Deposition

The porcine esophagi from recently slaughtered adult pigs were received as waste products from a local slaughterhouse (Khon Kean, Thailand). The mucosal epithelium was gently detached from the connective tissue layer, opened longitudinally, and rinsed with pH 7.4 phosphate buffer saline [25,26]. The esophageal mucosa with a thickness of 650 ± 30 μm was visually inspected for integrity and used as a surrogate for the permeation study.

The ex vivo permeation was measured using a modified Franz diffusion cell with a 2.01 cm^2^ diffusion area and 14.0 ± 0.2 mL receptor volume. The porcine mucosa used as a permeation membrane was securely clamped between the donor and receiver chambers, with the mucosa side facing the donor compartment. Then, 50% *v*/*v* ethanol was used as a receiver medium. The receiver compartment was set to 37 ± 0.5 °C and the hydrodynamics were maintained by stirring at 80 rpm with a digital magnetic stirrer (VELP SCIENTIFICA, Usmate Velate, Italy). The mounted mucosa was equilibrated with 1.5 mL of pH 6.8 SSF on the donor side for 15 min prior to initiating the experiment. Then, 10 mg of CBD in the form of the CBD liquisolid systems or the PM powder system was placed on the mucosal membrane at the donor chamber together with 1.5 mL of pH 6.8 SSF [23]. The donor compartment was tightly covered with paraffin film. At predetermined times (0, 1, 2, 3, 4, 5 and 6 h), a 0.8 mL receiver medium was withdrawn and an equal volume of pre-warmed fresh medium was restored. The obtained samples were dried at 45 °C using a vacuum concentrator (SpeedVac SPD300DDA, Thermo Scientific, Waltham, MA, USA) and the residue was redissolved with 0.2 mL methanol. The amount of permeated CBD was quantified by HPLC assay. 

*Permeation data analysis:* The cumulative mass of the permeated CBD was calculated and plotted against the time. The steady-state region of the permeation curve was used to compute the steady-state flux (*J_ss_*). The cumulative CBD permeated at 6 h (*Q_6h_*) was determined and used to calculate the permeation enhancement ratio (ER) as follows:

(4) $$J_{ss}=\frac{\Delta Q_{t}}{\Delta t\cdot A}$$

(5) $$Q_{t}=\frac{C_{t}\cdot V+\left(\sum_{t-1}^{t}C_{t-1}\right)\cdot V_{w}}{A}$$

(6) $$\mathrm{ER}\;=\frac{Q_{6h}\;of\;liquisolid\;system}{Q_{6h}\;of\;PM}$$

where *Q_t_* is the cumulative permeated amount of CBD and *C_t_* is the CBD concentration at the time point *t*. *C_t−_*_1_ is the CBD concentration at the previous time point. *V* refers to the total volume of the receiver medium, while *V_w_* is the withdrawn volume of receiver medium at each time point. A refers to the permeation surface area. ∆*Q_t_* is the difference in *Q_t_* between time points, and ∆t is the time difference [27].

For the deposition study, after 6 h of the permeation investigation, the mucosal membrane was separated and the remaining CBD powder was wiped out. The mucosa surface was washed with methanol (1 mL) and then deionized water (1 mL, 4 times). The mucosa area that was directly in contact with the CBD powder during permeation was separated, cut into small pieces using scissors and extracted thrice with methanol (2 mL). The methanolic extracts were collected, combined and dried under a vacuum at 45 °C using a vacuum concentrator (SpeedVac^®^ SPD300DDA, Thermo Scientific, Waltham, MA, USA). The dried residue was redissolved with 2.0 mL methanol and quantified by HPLC assay. The residual mucosa tissue was dried at 50 °C for 24 h and then weighed. The deposition amount of CBD per mucosa dry mass (µg/mg) was calculated.

### 2.6. HPLC Assay

The amount of CBD was assessed using a modified HPLC method [28]. An Agilent series 1260 combined with an Agilent ZORBAX Eclipse Plus column (C18, 100 × 4.6 mm, 3.5 μm) (Agilent Technologies, Inc., Santa Clara, CA, USA.) was used. The chromatographic separation was attained in 10 min using an isocratic protocol with a mixture of water:acetonitrile (30:70 *v*/*v*) comprising 0.1% formic acid set at a 1.5 mL/min flow rate. The injection volume was 20 µL. The column temperature was set at 45 °C, while the UV detector was set to 220 nm. The chromatographic condition demonstrated good linearity (R^2^ > 0.999) throughout the investigated concentration range (2–80 μg/mL).

### 2.7. Statistical Analysis

A one-way analysis of variance (ANOVA) with Tukey’s post hoc test operated via the SPSS program for Windows software (Version 17.0, Released 2008, Chicago, IL, USA: SPSS Inc.), was utilized to statistically analyze the data. The statistical significance was set as *p* < 0.05.

## 3. Results and Discussion

### 3.1. CBD Solubility in the Liquid Vehicles

Table 1 presents the solubility of the CBD in various liquid vehicles together with the liquid properties collected from the literature [29,30,31,32,33]. CBD is a hydrophobic compound; its solubility in water was found to be less than 0.005 mg/g (<0.005 mg/mL, considering the specific gravity value of water). The CBD was practically insoluble in glycerin, freely soluble in DEGEE, OM, PG, CM, PEG and P20, and very soluble in EtOH. According to the “like dissolves like” principle, the solubility of any solid compound in a liquid solvent varies and is influenced by the liquid properties, including the molecular structure and size, polarity, hydrophobicity/hydrophilicity and liquid viscosity [34,35]. For surfactant-type liquids (OM, CM and P20), the solubility of CBD decreased with the liquid hydrophilicity, as indicated by the hydrophilic–lipophilic balance (HLB) values, and with the viscosity. In the case of low-polarity solvent-type liquid vehicles (EtOH, DEGEE, PEG and glycerin), the CBD solubility was also correlated with the solvent hydrophobicity and solvent viscosity. The highest solubility of CBD in EtOH and DEGEE may be attributable to the low polarity, as indicated by the dielectric constant values, with the lowest viscosity as compared to PG, PEG and glycerin, respectively. 

Concerning the solubility of CBD in the liquid vehicles, five vehicles, namely, EtOH, DEGEE, OM, PG and CM, were selected for further CBD liquisolid system investigations. These liquid vehicles are the common liquids used in oral, parenteral, topical, transdermal and mucosal preparations. Additionally, their solubilization, as well as their permeation enhancing capabilities for a number of active ingredients, have been reported [31,32,37,38,39].

### 3.2. L_f_ of the CBD Solutions

The L_f_ values of each CBD solution were investigated directly by determining the amount of MCC in the MCC–CSD required to convert the CBD solution into free-flowing powder. As shown in Table 2, the L_f_ of the investigated vehicles ranged from 0.181 ± 0.024 to 0.145 ± 0.016, with DEGEE yielding the lowest value. Consequently, the amount of MCC–CSD needed was highest for the DEGEE-based liquisolid formulation when compared to the others. It has been reported that the liquid viscosity influences its capacity to be absorbed/adsorbed onto the carrier–coating material. The lower-viscosity liquid exhibits a higher quantity of liquid diffusion throughout the MCC–CSD powder due to the faster rate of penetration onto and into the surface and capillary pores, with a lower possibility of pore-clogging [3,40]. The lower L_f_ value of the lower-viscosity liquid vehicles on the MCC–CSD powder is in line with results presented in other reports [3,18,34].

### 3.3. CBD Liquisolid System Characteristics

Five formulations of CBD liquisolid powders were prepared based on different liquid vehicles. To exclude the effect of the L_f_ value on the required amount of MCC–CSD, an L_f_ value equal to 0.145 and a CBD concentration in liquid vehicles of 20% *w*/*w* were used. All of the resulting CBD liquisolid powders were white, dry-looking and non-adherent. Their good and acceptable flowability was confirmed by the flowability tests, specifically by the flow rate, angle of slide and angle of repose, respectively. As depicted in Table 2, the flow rate through an orifice was above 6 cm^3^/s with angle of repose values of less than 35°, which were classified as good flow properties [36]. The angle of slide was under the acceptable value (≤33°). The angle of slide is considered an efficient flowability test method and is widely used to assess the flowability of liquisolid powder [21,41].

#### 3.3.1. Intermolecular Interaction by FTIR

ATR-FTIR was used to assess the interaction between the CBD and excipients in the liquisolid powder. The spectrum of CBD exhibits characteristic bands with the maximum at 3517 and 3406 (O–H stretching), bands in the range of 3100–2800 (asymmetric and symmetric C–H stretching), two bands at 1623 and 1581 (C=C stretching), and bands at 1373 (C–H bending) and 1214 cm^−1^ (C–O stretching), respectively [12,42]. 

**Figure 1:**
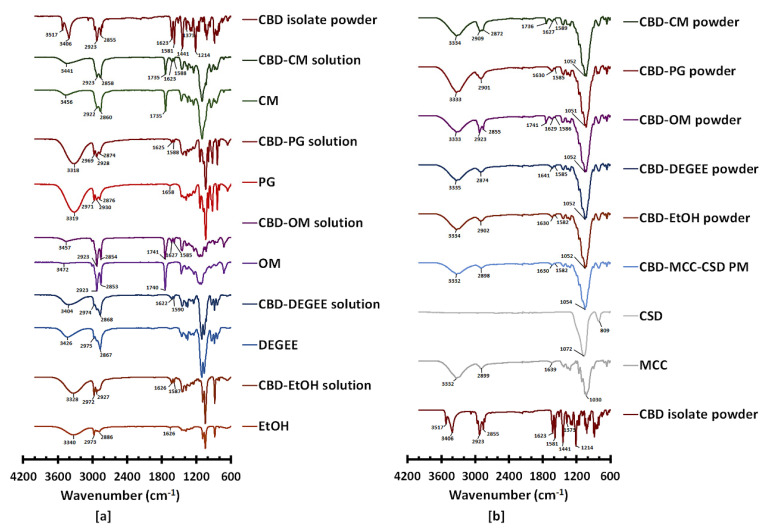
ATR-FTIR of (**a**) the CBD isolate, CBD–liquid vehicle solutions (20% *w*/*w*) and pure liquid vehicles; (**b**) the CBD liquisolid powder and MCC, CSD and PM powders.

The FTIR spectra of the CBD–liquid vehicle solutions are shown in Figure 1a. The O–H stretching band of EtOH, OM and CM underwent a redshift, with an increased intensity when solubilized by CBD. For the CBD–DEGEE solution, a shift in the O–H stretching band from 3426 to 3404 cm^−1^ with comparable intensity was observed, whereas in the case of the CBD–PG solution, a decrease in the O–H stretching band intensity was observed. Additionally, shifts in the characteristic CBD bands at 1581 and towards the higher wavenumbers at 1589, 1590, 1585, 1588 and 1588 cm^−1^ were observed for the CBD–EtOH, CBD–DEGEE, CBD–OM, CBD–PG and CBD–CM solutions, respectively. These phenomena suggest that a solute–solvent interaction occurred between the CBD and liquid vehicles. The solvation between the solute and solvent neutral molecules is generally associated with the weak attractions, known as Van der Waals forces (Keesom, Debye and London forces, as well as hydrogen bonds) [43]. The modification of the CBD C=C stretching band at 1581 cm^−1^ caused by the association of this moiety with another molecule has previously been reported [12].

Figure 1b presents the FTIR spectra of the CBD liquisolid powder in comparison with those of the PM, MCC and CSD. For the PM powder, the superposition of the FTIR spectra of CBD, MCC and CSD characteristic bands indicates the absence of the intermolecular interaction between these compounds when they were physically mixed. For the CBD liquisolid powder, only those prepared with nonvolatile vehicles exhibited shifts in the CBD C=C stretching band. This band was repositioned to 1586, 1586, 1585 and 1589 cm^−1^ for CBD–DEGEE, CBD–OM, CBD–PG and CBD–CM, respectively. This suggests that the CBD might still have been in the solubilized form, with a liquid vehicle, when deposited onto the MCC–CSD surface.

#### 3.3.2. Solid State and Morphological Characteristics

**Figure 2:**
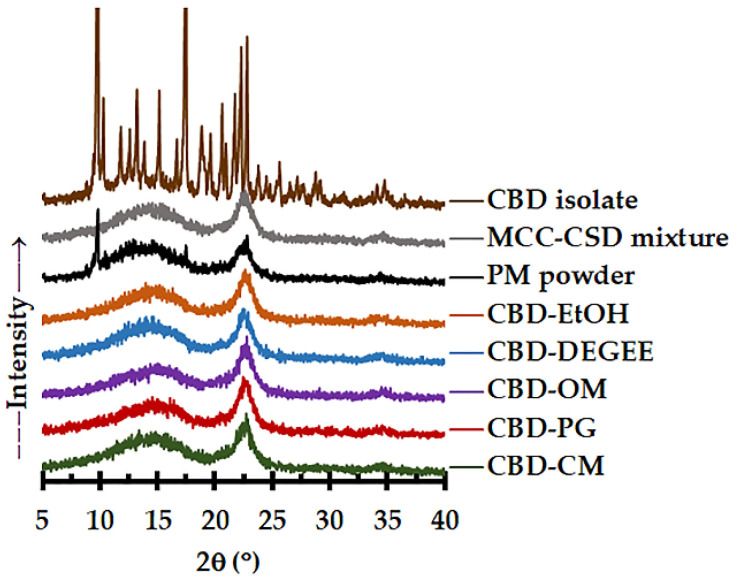
XRD diffractograms of the CBD isolate, MCC–CSD mixture, PM powder and CBD liquisolid powder.

The solid state of the CBD in the CBD liquisolid systems was investigated using an XRD study. As presented in Figure 2, the CBD isolate possesses a crystalline structure, as demonstrated by a sequence of sharp diffraction peaks between 5 to 40° [12,42]. The PM powder showed the specific patterns of the CBD (diffraction peaks at 9.8° and 17.5°), MCC (strong sharp peak at 22.6°) and CSD (halo pattern). For the CBD liquisolid powder, irrespective of the liquid vehicles, the characteristic peaks of the CBD disappeared. This indicates that the CBD was presented in the non-crystalline state. The amorphization and absence of crystallinity of the active ingredients caused by the liquisolid technique are in line with the results presented in other reports [18,41].

**Figure 3:**
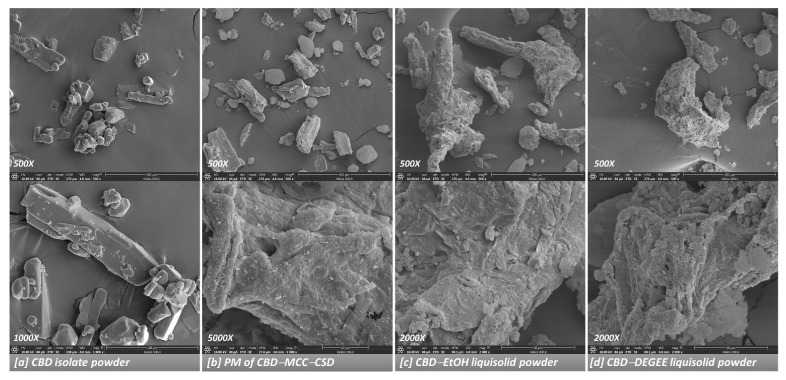
FESEM images of the CBD isolate (**a**), PM powder (**b**) and CBD liquisolid powder (**c**,**d**).

The surface morphology of the CBD liquisolid powder was characterized via FIB-FESEM, as shown in Figure 3. The CBD isolate presented as irregular-shaped crystalline particles of various sizes with well-defined edges, with a superposition of the small crystals onto the larger ones. In the PM powder, the presence of the CBD isolate, together with the micron-sized cellulosic fibril MCC particles surface-coated with CSD particles, was observed. For the CBD liquisolid powder, the disappearance of the CBD isolate was observed. This result is in line with the XRD information, indicating the absence of CBD crystallinity. It should be emphasized that no visible difference could be seen between the CBD liquisolid powders prepared using different liquid vehicles (Figure 3c,d and Supplementary Materials, Figure S1).

#### 3.3.3. In Vitro Release 

**Figure 4:**
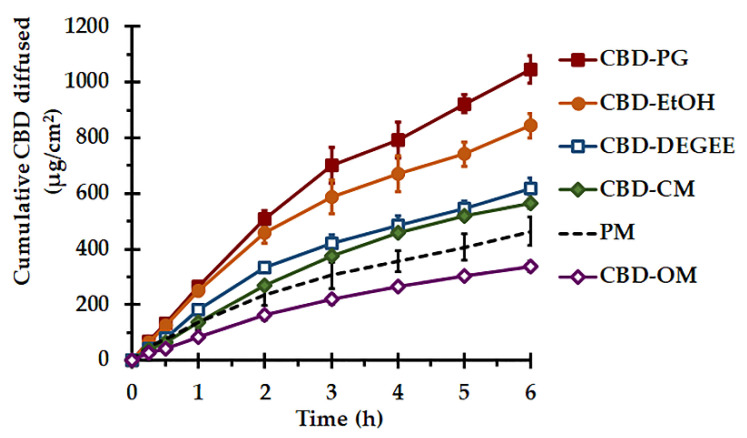
In vitro release performance, in terms of diffusion, of the CBD liquisolid systems and PM under sink conditions at 37 ± 0.5 °C (mean ± SD, *n* = 3).

The CBD release was investigated in terms of in vitro diffusion, using the vertical diffusion cells with 50% EtOH as a receiver medium. The solubility of the CBD in 50% EtOH was found to be sufficient for obtaining the sink condition throughout the experiment [44]. The porous synthetic membrane was used to support and separate the CBD liquisolid systems or PM from the receptor fluid due to its minimal diffusion resistance, as a rate-limiting diffusion barrier [45]. The cumulative amounts of CBD diffused as per the surface area versus the time plots are presented in Figure 4, and their diffusion parameters are presented in Table 3. Statistically significant differences were found between these parameters (*p* < 0.05). To determine the kinetics of the CBD transport from the liquisolid systems, the diffusion profiles were analyzed with zero-order and Higuchi models. The zero-order model characterizes a constant rate of CBD release from the systems, regardless of the CBD concentration, while the Higuchi denotes the release process based on Fickian diffusion. As presented in Table 3, it can be seen that the Higuchi model yielded the regression coefficient, R^2^, close to 1. The higher linearity of the Higuchi model with greater R^2^ values than that of the zero-order model indicated that the CBD diffusion kinetics followed a Fickian diffusion process. According to Fick’s law, the rate of diffusion of CBD over a specific surface area is in proportion to the concentration gradient. The rate of release decreases with the decrease in the concentration gradient, caused by the increased diffusion path [24]. The rate of CBD diffusion from the CBD–PG liquisolid system was the fastest, and it was 2.4 times faster than that of the PM system, while the CBD–OM yielded the slowest CBD diffusion rate (*p* < 0.05). 

The better in vitro release performance of the CBD liquisolid formulations, namely CBD–PG, CBD–EtOH, CBD–DEGEE and CBD–CM, than that of PM was associated with the enlargement of the CBD surface area. This resulted from the adsorption/absorption of the CBD onto the MCC–CSD surface in a non-crystalline state, as confirmed by the XRD study. Additionally, in nonvolatile liquid-based liquisolid systems, the wettability and saturated solubility of CBD in the microenvironment might also be modulated [14,18,46,47]. 

The process of the CBD release involves the penetration of the MCC–CSD pores by the release medium, the dissolution of CBD by the medium and the diffusion/transfer of the dissolved CBD [18]. To study the release through the diffusion cells, a small volume of SSF was added to the donor compartment. Under this condition, nonvolatile vehicles that adsorbed/absorbed onto the MCC–CSD the together with CBD appear to play a remarkable role. It could be speculated that a nonvolatile vehicle acts as a cosolvent and modulates the solubility of CBD in the microenvironment of the diffusion layer between the liquisolid particle/release medium interfaces [14,47]. In essence, the vehicles that exhibit a high solvency capacity for CBD should provide a high CBD release. Nevertheless, the viscosity, hydrophilicity (dielectric constant, HLB) and micellization capacities of vehicles appear to play significant roles in the drug release [14,41,46]. As presented in the solubility study, DEGEE, OM and PG had comparable solvency capacities to CBD, while the lower diffusion efficiency of CBD–OM compared to those of the CBD–DEGEE and CBD–PG liquisolid systems, by a 2.6–3.1-fold difference, is related to the amphiphilic structure and highly viscous nature of OM. A recent investigation revealed that the hydration of viscous excipients, such as amphiphile liquids, in the presence of a small aqueous medium volume results in the viscosity increment. Such a rheological change upon hydration could give rise to the pore-clogging and locking of the CBD–liquid vehicle inside the carrier–coating pores, eventually limiting the CBD release [41]. The entrapment of the drug inside the aggregates of the surfactant molecules, known as micelles, might also be related to the release retardation [46]. Additionally, the low HLB value, which indicates the less hydrophilic nature of the OM molecule (Table 1), may also limit the extent of desorption of the liquid medicament on the MCC–CSD surface. According to Van Speybroeck et al. [48] and Williams et al. [49], the incomplete desorption of lipid-based formulations may be associated with the lipophilicity and viscosity of nonvolatile liquids. The drug release retardation from the surfactant-based liquisolid system, as compared to the low-polarity solvents, is in line with the results presented in a previous reports [46].

The highest diffusion rate and efficiency of the CBD–PG liquisolid system, among all the systems examined, is attributable to the CBD solubilizing capacity and suitable viscosity of PG. It might be postulated that PG could efficiently promote CBD solubilization at the microenvironment level. Because of the more viscous nature of PG compared to that of DEGEE, it is quite possible that PG could diffuse out of the MCC–CSD particle at a slower rate, and thus a relatively minute quantity of PG remains, acting sufficiently as a cosolvent at the stagnant diffusion layer.

In the case of the CBD–EtOH liquisolid system, EtOH was used to dissolve and promote the CBD loading. As a volatile liquid, it was removed during the manufacturing process completely, leaving the non-crystalline CBD deposited onto the MCC–CSD surface. The release enhancement of the CBD by the CBD–EtOH liquisolid system is solely caused by the increase in the available surface area of the CBD. The better diffusion performance of CBD–EtOH compared to that of nonvolatile liquid-based liquisolid systems is probably attributable to the larger CBD surface area. Because of the low viscosity and surface tension of EtOH [32], the greater and even distribution of the CBD–EtOH solution throughout the MCC–CSD surface and pores could be achieved efficiently.

#### 3.3.4. Ex Vivo Permeation and Deposition

The permeation and deposition of the CBD liquisolid systems and PM were comparatively assessed using vertical diffusion cells in occlusive conditions. Mucosa obtained from porcine esophagus was used as a substitute for oral tissue, owing to the comparability in the structure, lipid composition, and permeability characteristics between porcine esophageal and buccal mucosae [45,46]. It has been reported that the histological and constitutional characteristics of porcine esophageal and buccal mucosa are comparable with the analogous human mucosa [50,51]. Buccal and esophagus mucosa are covered by a stratified squamous and non-keratinized epithelium with a lipid composition. The permeability barrier in these tissues is formed of groups of lipid lamellae positioned in the intercellular spaces of the superficial epithelial layer [50,51,52,53,54]. Additionally, porcine esophageal mucosa provides experimental benefits, including an even membrane thickness, a high yield of usable mucosa, and simplicity of preparation [27,55]. The utilization of porcine esophageal mucosa as a surrogate for a nonkeratinized mucosal membrane for the purpose of ex vivo permeation studies has been widely reported [25,26,27,49,55,56,57]. A 50% EtOH solution was used as a receiver medium to maintain the sink conditions. According to Casirahi et al. [44], the solubility of CBD in 50% EtOH was found to be 2.2 ± 0.1 mg/mL. This solubility value was 733-fold greater than that in pH 6.8 SSF (<0.005 mg/mL). The CBD solubility in 50% EtOH was considered to be sufficient for 10 mg of CBD, in the form of the CBD liquisolid systems or the PM powder system, to maintain the sink conditions throughout the experiment. Moreover, 50% EtOH has been used as a receiver fluid in permeation investigations of very hydrophobic drugs, including cannabinoids [44,58,59].

**Figure 5:**
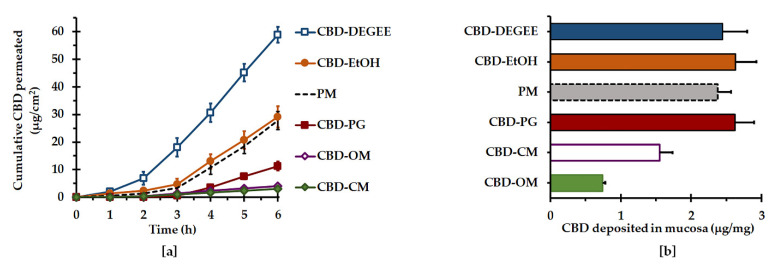
Ex vivo mucosal permeation (**a**) and deposition (**b**) of the CBD liquisolid systems and PM at 37 ± 0.5 °C (mean ± SD, *n* = 4).

The cumulative amounts of CBD permeated across the mucosa vs. time and the permeation parameters are illustrated in Figure 5a and Table 4, respectively. Surprisingly, the CBD liquisolid systems resulted in either permeation enhancement or retardation, depending on the type of liquid vehicle. The permeation profiles of CBD were sigmoidal in nature, composed of an initial slow-permeation phase followed by a linear faster-permeation phase. The data acquired from the latter permeation phase were optimally fitted to a zero-order model, with a high coefficient of determination (0.992 ± 0.008 to 0.999 ± 0.002). This indicated that the rate-limiting process of permeation by the CBD liquisolid systems was the permeation through mucosal membrane instead of the CBD diffusion from the liquisolid system. There were significant differences in the permeation flux and Q_6h_ between the CBD liquisolid formulations. CBD–DEGEE resulted in the highest flux and Q_6h_ values compared with the others (*p* < 0.05). When compared to the control PM system, CBD–DEGEE provided an enhancement ratio of 2.1 folds, while the surfactant-based liquisolid systems retarded the CBD permeation by approximately 2.5 folds. 

The mucosal deposition results of the CBD liquisolid systems are presented in Figure 5b. It was found that the CBD–EtOH, CBD–PG and CBD–DEGEE liquisolid systems and the PM yielded comparable amounts of CBD deposited in the permeation mucosa, ranging from 2.3 ± 0.2 to 2.6 ± 0.3 μg per 1 mg of dry mucosa. On the other hand, CBD–CM and CBD–OM exhibited significantly lower CBD depositions, by 1.5 and 3.2 folds, respectively, compared to that of the PM.

CBD is a highly lipophilic molecule, with a calculated log K_ow_ of ~8, and its permeation was assumed to be a passive diffusion through a transcellular pathway. Recent investigations revealed that a small amount of CBD was able to permeate through mucosa or skin, while a finite amount was deposited inside the permeated membrane [7,44,60]. Similar to transdermal delivery, transmucosal delivery is a complex event involving drug release from the system, the drug’s partition into and diffusion across mucosa, and the permeation enhancement of the delivery system. The best CBD permeation performance, exhibited by CBD–DEGEE, was associated with the permeation-enhancing property of DEGEE. A proposed mechanism of DEGEE in promoting the drug permeation throughout the biological membrane may relate to its ability to pass into and be deposited in it, modifying the drug solubility in the membrane and improving the drug’s partition into it. Moreover, it has been pointed out that DEGEE might incorporate itself into the lipid bilayers, resulting in intercellular lipid fluidization [31,61]. DEGEE is a powerful permeation enhancer with an excellent solubility enhancement ability. Its permeation enhancement capacity when applied to several lipophilic drugs, including CBD, has been demonstrated [31,62,63]. 

The CBD–EtOH liquisolid system and PM had comparable CBD permeation and deposition performances, despite the fact that they showed significant differences in the CBD diffusion rate. These results confirm that the permeation of CBD through the mucosa was the rate-controlling process. Increasing the CBD release rate and amount via an increased surface area did not affect the CBD permeation performance. An interesting result was also found for the CBD–PG liquisolid system. Even though CBD–PG presented with the fastest CBD diffusion, its permeation flux and Q_6h_ values were much lower than those of the control. The high solubility of CBD in PG indicates the high affinity of CBD to this vehicle, which might lead to a decrease in the thermodynamic activity, and thus CBD permeation. Thermodynamic activity is considered to be a driving force of the drug partitioning process into the biological membrane. The drug partition coefficient between the vehicle and biological membrane typically decreases with the drug solubility in the vehicle [64].

The retardation effects on the CBD permeation and deposition by the CBD–CM and CBD–OM liquisolid systems are attributable to the formation of micelles, entrapping the CBD molecule inside. It is generally considered that surfactant molecules aggregate spontaneously when their concentration in an aqueous medium is above the critical micelle concentration. The resulting self-assembly particles, so-called micelles, are in the colloidal size range, which may have a lower partition into the mucosal membrane. Additionally, the use of surfactants in mucosal intercellular lipid extraction has been reported [38,64,65]. This might entail a negative effect on the permeation of lipophilic drugs, such as CBD. It was found that surfactants, at low concentrations, could only promote the permeability of hydrophilic compounds that pass through the biological membrane by the paracellular route. Several investigations suggested that the permeation-enhancing capacity of surfactants through epithelial membranes, including the mucosa, relies upon the physicochemical properties of the permeating drugs, especially the lipophilicity and the permeation pathway of that compound [38,65].

## 4. Conclusions

This investigation presented the mucosal permeation and deposition capacities of the CBD by liquisolid systems for the first time. Different liquid vehicles exhibited distinct influences on the ex vivo mucosal permeation and deposition, as well as the in vitro release of CBD by the liquisolid systems. Interestingly, the enhancement of the CBD release appears to have no beneficial effect on the CBD permeation and deposition performance. As in the case of CBD–EtOH liquisolid systems and the control PM, their comparable CBD permeation and deposition was evident, although CBD–EtOH exhibited a 1.9-fold increment in the CBD diffusion rate and efficiency. This is probably related to the fact that the permeation of CBD through the mucosal membrane is the rate-limiting process. The CBD–PG liquisolid system yielded the best CBD release behavior, yet it retarded the CBD permeation. Additionally, surfactant-based liquisolid systems greatly retarded the CBD permeation and deposition. On the other hand, the CBD–DEGEE liquisolid system significantly promoted the permeation of CBD across the mucosa. These findings strongly support the notion that the liquid vehicles used in liquisolid systems can promote or suppress mucosal permeation and deposition. This study discloses that, by using suitable liquid vehicles with a permeation-enhancing capacity, the application of liquisolid systems to transmucosal delivery is feasible. The combination of liquisolid powders with suitable components, e.g., mucoadhesive polymers, can offer a promising alternative transmucosal delivery system. The ability of the CBD–DEGEE liquisolid system to deliver CBD across the mucosa underlines the potential of this system for transmucosal delivery applications. Further investigations concerning the development of liquisolid-based mucoadhesive delivery systems, e.g., mucoadhesive tablets, for CBD should be performed.

## Figures and Tables

**Table 1:** Solubility of the CBD in liquid vehicles at 37 ± 0.5 °C.

Liquid Vehicles					CBD Solubility (mg/g)
Types	Dielectric Constants *	HLB **	Viscosity (mPa⋅s) ***	Specific Gravity ****	
EtOH	24.3	N/A	1.2	0.814 ± 0.002	>1000
DEGEE	14.1	N/A	4.8	0.969 ± 0.002	579.46 ± 11.79 ^a^
OM	N/A	9.0	75–95	0.924 ± 0.001	540.31 ± 4.59 ^a^
PG	32	N/A	58	1.027 ± 0.002	521.04 ± 55.42 ^a^
CM	N/A	12.0	80–110	1.041 ± 0.002	386.77 ± 48.85 ^b^
PEG	12.5	N/A	105–130	1.120 ± 0.003	342.18 ± 49.90 ^b^
P20	N/A	16.7	400	1.084 ± 0.002	261.73 ± 39.33 ^c^
Glycerin	40.1	N/A	1490	1.264 ± 0.003	0.09 ± 0.03 ^d^
Deionized water	78.5	N/A	1	0.998 ± 0.002	<0.005

Mean ± SD, *n* = 3; ^a–d^ means in the same column sharing a common superscript letter are not different (*p* > 0.05), as analyzed by one-way ANOVA and Tukey’s post-hoc test. N/A = not applicable. * Dielectric constant values at 25 °C, adapted from [30,31]. ** HLB, hydrophilic–lipophilic balance values, adapted from [32]. *** Dynamic viscosity values at 20 °C, adapted from [29,31,32,33]. **** Specific gravity at 25 °C determined by pycnometer [36].

**Table 2:** Liquid load factor and flowability of the CBD liquisolid powder formulations.

Formulations	L_f_ *	Flow Rate (cm^3^/s)	Angle of Slide (°)	Angle of Repose (°)	Flowability **
CBD–EtOH	N/A	6.86 ± 0.37	29.00 ± 0.82	31.01 ± 0.65	Good
CBD–DEGEE	0.145 ± 0.016	6.31 ± 0.26	32.00 ± 0.82	33.02 ± 0.63	Good
CBD–OM	0.153 ± 0.027	7.04 ± 0.03	30.00 ± 0.01	31.80 ± 1.34	Good
CBD–PG	0.181 ± 0.024	8.99 ± 0.45	29.33 ± 0.47	33.53 ± 0.81	Good
CBD–CM	0.178 ± 0.017	8.54 ± 0.63	29.67 ± 0.47	33.10 ± 0.61	Good

Mean ± SD, *n* = 3. N/A = not applicable. * L_f_ or liquid load factor denotes the quantity ratio of CBD solution (20% *w*/*w*) in a nonvolatile vehicle to the MCC required to transform that CBD solution into the free-flowing CBD liquisolid powder having a ≥6 cm^3^/s flow rate. ** Flowability determined from the angle of repose value as per the United States Pharmacopeia criteria [36].

**Table 3:** In vitro release parameters, in terms of diffusion, of the CBD liquisolid systems and PM.

Formulations	Zero Order		Higuchi		*Q*_6*h*_ (µg)	Diffusion Efficiency (%)
	R^2^	K_0_ (µg·cm^−2·^min^−1^)	R^2^	K_H_ (µg·cm^−2^⋅min^−1/2^)		
PM system	0.974 ± 0.012	71.3 ± 7.2	0.996 ± 0.003	214.6 ± 22.4 ^a^	930.5 ± 52.7 ^a^	5.63 ± 0.74 ^a^
Liquisolid systems						
CBD–EtOH	0.951 ± 0.008	133.3 ± 14.5	0.994 ± 0.003	405.4 ± 43.0 ^b^	1696.7 ± 193.0 ^b^	10.51 ± 0.92 ^b^
CBD–DEGEE	0.951 ± 0.012	93.8 ± 7.4	0.993 ± 0.003	285.3 ± 21.8 ^c^	1241.3 ± 76.3 ^c^	7.56 ± 0.39 ^c^
CBD–OM	0.975 ± 0.015	55.6 ± 2.0	0.995 ± 0.002	167.0 ± 6.9 ^a^	669.0 ± 17.5 ^d^	4.05 ± 0.32 ^d^
CBD–PG	0.968 ± 0.013	169.6 ± 6.1	0.993 ± 0.004	511.3 ± 20.2 ^d^	2102.7 ± 68.8 ^e^	12.45 ± 0.42 ^e^
CBD–CM	0.972 ± 0.004	95.4 ± 3.1	0.994 ± 0.002	287.3 ± 9.2 ^c^	1138.4 ± 49.6 ^c^	6.86 ± 0.27 ^c^

Mean ± SD, *n* = 3; ^a–e^ means in the same column without a common superscript letter are different (*p* < 0.05), as analyzed by one-way ANOVA and Tukey’s post hoc test. *K*_0_ and *K_H_* refer to the zero-order and Higuchi diffusion rates (0.25–6 h), respectively. *R*^2^ is the coefficient of determination calculated from a set of diffusion values over 0.25–6 h. *Q*_6*h*_ (µg) is the cumulative CBD diffused at 6 h. Diffusion efficiency (%) is the percentage of the rectangle area under the diffusion curve up to 6 h.

**Table 4:** Ex vivo permeation parameters of the CBD liquisolid systems and PM system.

Formulations	*J_ss_* (µg·cm^−2^·h^−1^)	*Q*_6*h*_ (µg)	ER
PM system	8.09 ± 0.67 ^a^	55.83 ± 6.46 ^a^	N/A
Liquisolid systems			
CBD–EtOH	8.10 ± 0.59 ^a^	58.54 ± 7.77 ^a^	1.05
CBD–DEGEE	13.68 ± 0.74 ^b^	118.38 ± 5.79 ^b^	2.12
CBD–OM	0.89 ± 0.11 ^c^	8.27 ± 0.83 ^c^	0.15
CBD–PG	3.65 ± 0.51 ^d^	22.66 ± 3.23 ^d^	0.41
CBD–CM	0.68 ± 0.11 ^c^	6.18 ± 1.42 ^c^	0.11

Mean ± SD, *n* = 4. ^a–d^ means in the same column without a common superscript letter are different (*p* < 0.05), as analyzed by one-way ANOVA and Tukey’s post hoc test. *J_ss_* is the steady-state permeation flux (µg·cm^−2^·h^−1^) calculated over 3–6 h. *Q*_6h_ is the cumulative CBD permeated at 6 h (µg). ER refers to the enhancement ratio of the cumulative CBD permeated at 6 h, calculated based on the PM formulation. N/A = not applicable.

## Data Availability

Data available on request.

## Supplementary Materials

The following supporting information can be downloaded at: https://www.mdpi.com/article/10.3390/pharmaceutics14091787/s1, Figure S1: FESEM images of CBD liquisolid powder prepared with nonvolatile vehicles (PG, OM and CM).
