# Supplementary figures and images for: In Vitro Release, Mucosal Permeation and Deposition of Cannabidiol from Liquisolid Systems: The Influence of Liquid Vehicles
Source: Pharmaceutics. 2022 Aug 26;14(9):1787. doi: 10.3390/pharmaceutics14091787 (PMC9503133; doi:10.3390/pharmaceutics14091787)

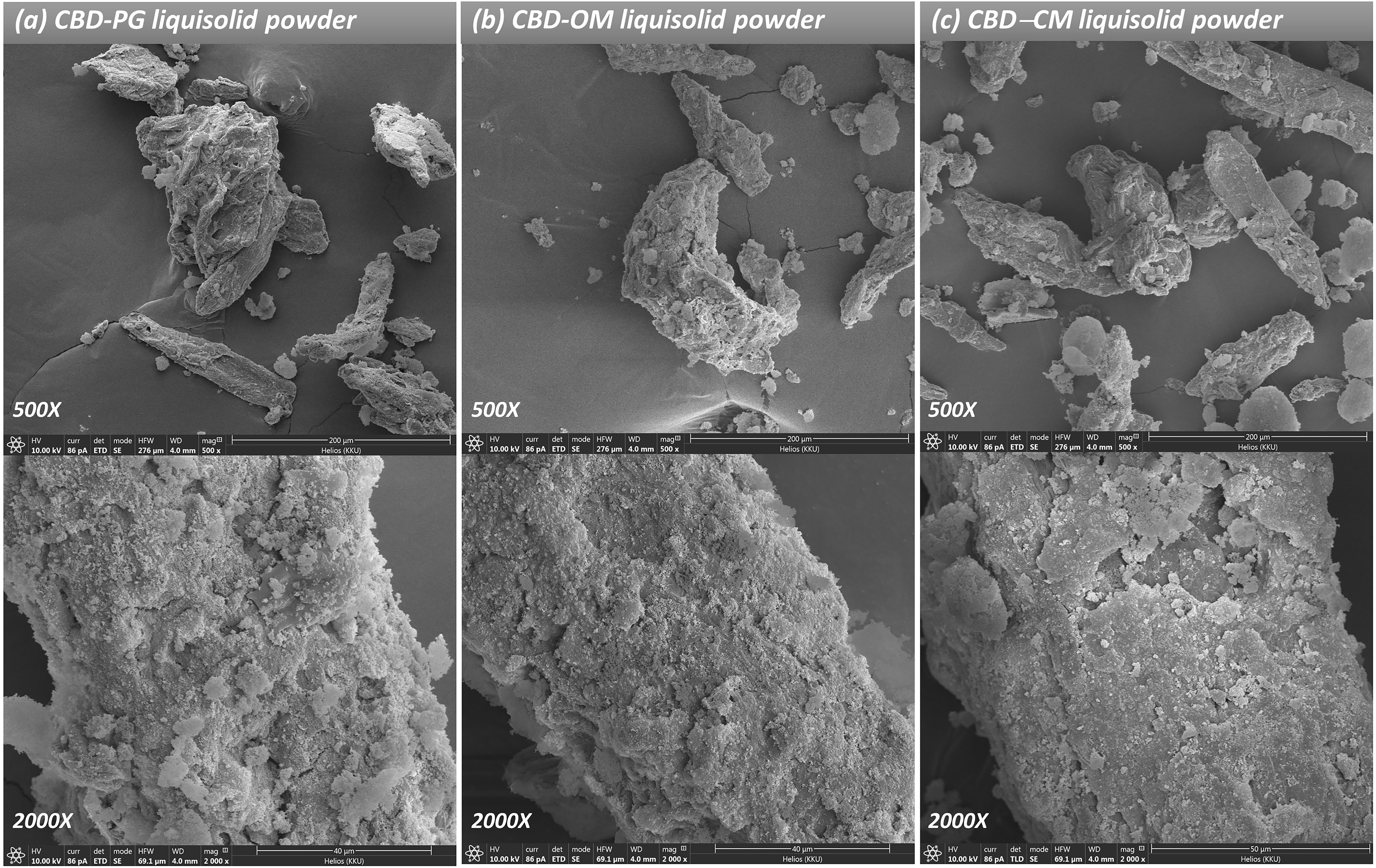

Supplement: Supplementary file 1 [file pharmaceutics-14-01787-s001.zip › pharmaceutics-1851363-supplementary.tif]
